# Interface Microstructure and Mechanical Properties of Al/Steel Bimetallic Composites Fabricated by Liquid-Solid Casting with Rare Earth Eu Additions

**DOI:** 10.3390/ma15196507

**Published:** 2022-09-20

**Authors:** Feng Mao, Po Zhang, Shizhong Wei, Chong Chen, Guoshang Zhang, Mei Xiong, Tao Wang, Junliang Guo, Changji Wang

**Affiliations:** 1 National Joint Engineering Research Center for Abrasion Control and Molding of Metal Materials, Henan University of Science and Technology, Luoyang 471003, China; 2 Longmen Laboratory, Luoyang 471000, China; 3 School of Materials Science and Engineering, Henan University of Science and Technology, Luoyang 471003, China

**Keywords:** Al/steel bimetal, bonding, Eu, compound casting, EBSD, EPMA

## Abstract

To improve the Al/Steel bimetallic interface, Eu was firstly added to the Al/Steel bimetallic interface made by liquid-solid casting. The effects of Eu addition on the microstructure, mechanical capacities, and rupture behavior of the Al/Steel bimetallic interface was studied in detail. As the addition of 0.1 wt.% Eu, the morphology of eutectic Si changed from coarse plate-like to fine fibrous and granular in Al-Si alloys, and the average thickness of the intermetallic compounds layer decreased to a minimum value of 7.96 μm. In addition, there was a more sudden drop of Fe in steel side and the Si in Al side was observed to be more than the other conditions. The addition of Eu did not change the kinds of intermetallic compounds in the Al/steel reaction layer, which was composed of Al_5_Fe_2_, τ_1_-(Al, Si)_5_Fe_3_, Al_13_Fe_4_, τ_5_-Al_7_Fe_2_Si, and τ_6_-Al_9_Fe_2_Si_2_ phases. The addition of the element Eu did not change the preferential orientation of the Al_5_Fe_2_, τ_1_-(Al, Si)_5_Fe_3_, Al_13_Fe_4_, τ_5_-Al_7_Fe_2_Si, and τ_6_-Al_9_Fe_2_Si_2_ phases, but refined the grain size of each phase and decreased the polar density of Al_5_Fe_2_ phase. Eu was mainly enriched in the front of the ternary compound layer (τ_6_-Al_9_Fe_2_Si_2_) near the Al side and steel matrix. The Fe and Al element distribution area tended to narrow in the interface after the addition of 0.1 wt.% Eu, which is probably because that Eu inhibits the spread of Al atoms along the c-axis direction of the Al_5_Fe_2_ phase and the growth of Al_13_Fe_4_, τ_5_-Al_7_Fe_2_Si, and τ_6_-Al_9_Fe_2_Si_2_ phases. When the Eu content was 0.1 wt.%, the shear strength of the Al/Steel bimetal achieved a maximum of 31.21 MPa, which was 47% higher than the bimetal without Eu.

## 1. Introduction

In recent years, Al/steel bimetallic has been widely studied [1,2,3,4,5,6] because it combines the excellent characteristics of aluminum alloy and steel [7,8,9,10,11,12,13,14], such as high intensity of steel and lower density of aluminum alloy. Therefore, it is widely used in the field of fuel economy and is lightweight. The liquid-solid composite method is a frequent method for the preparation of Al/Steel bimetallic composites because of its simple preparation process and low requirements in material selection. Due to the huge difference in chemical and physical properties of aluminum and steel, the poor wettability of liquid aluminum to steel and the easy oxidation of steel are major challenges for Al/Steel composite casting [15]. However, the formation of brittle intermetallic compounds (Al_5_Fe_2_) in the reaction region is another difficulty for Al/steel composites [16,17,18].

Current research has focused on improving the Al/steel bimetallic interface by adding different types of intermediate coatings between aluminum and steel to prevent the formation of Al-Fe intermetallic compounds. Jiang et al. [19,20,21] found that hot-dipped aluminum or zinc plating on the steel surface could improve the wettability of liquid aluminum on the steel surface, which can enhance the bonding strength of the Al/Steel bimetallic interface. Some scholars have tried to decrease the thickness of the brittle intermetallic phase of Al/steel bimetals by adding alloying elements to the hot-dipped aluminum melt. Cheng et al. [22] investigated the effect of hot dip coating of aluminum alloys with different Si contents on the intermetallic compound layer. The results indicated that the solid solution of Si in the Al_5_Fe_2_ phase increases with the increase of Si content. The Si atoms occupied their vacant position on the C-axis of the Al_5_Fe_2_ phase forming a ternary Al-Fe-Si intermetallic (Al_7_Fe_2_Si), which reduced the spread rate of Al atoms to Al_5_Fe_2_ and caused a gradual decrease in the thickness of Al_5_Fe_2_. Chen et al. [23] found that the addition of Ni element into the hot dip coating of aluminum alloy melt could reduce the growth rate of the Al_5_Fe_2_ layer, which effectively controlled the growth of Al_5_Fe_2_ hard and brittle phase.

Rare earth elements have long been used to refine and strengthen alloys, which can modify the eutectic Si and refine the α-Al grains in Al-Si alloys [24,25,26,27]. Li et al. [28] investigated the variation of eutectic Si in A356 alloys with various Eu contents. The eutectic Si morphology of A356 alloys was transformed from flakes to fibers with the increase of Eu content, which substantially enhanced the mechanical properties. Muhammad et al. [29] showed that at the Sc content of 0.4 wt.% Sc, the eutectic Si in the A357 alloy changed from acicular to fibers and the grain size in the alloy was reduced by 80%, which make the ultimate tensile strength increase by 28%. Shi et al. [30] showed that the addition of 0.3 wt.% Er had a significant influence on the refinement of α-Al grains and the morphological changes of eutectic Si. Li et al. [31] indicated the effect of adding different contents of Y on the microstructure of Al-7Si-0.5 Mg alloy. It was found that the eutectic Si morphology changed from sheet-like to fine-branched, and the tensile properties were significantly enhanced. From previous research work, the study of RE modification has mainly focused on a single alloy; there are very few studies on the influence of RE addition on the Al/steel bimetallic interface.

Eu was the only rare earth element capable of producing fibrous eutectic Si in Al-Si alloy, which maintains similarity with Sr and Na. However, the effect of rare earth Eu on the interface microstructure and mechanical properties of Al/steel bimetallic composites has not yet been widely investigated. In this paper, rare earth element Eu was first added to the liquid-solid composite of Al/steel bimetal, and the interface microstructures of Al/Steel bimetallic composites and the effect mechanism was studied by SEM, EPMA and EBSD. Then, the effect of Eu on the mechanical capacities and rupture behavior of the Al/steel bimetallic interface was also studied. Many results are firstly reported and are very novel.

## 2. Experimental

### 2.1. Materials

The Al-7Si alloys with different Eu contents alloy and 45 steel were respectively selected as casting alloys and solid matrix materials to produce Al/Steel bimetallic, and the Al-7Si alloys with different Eu contents were also used as hot-dip aluminum plating materials for 45 steel matrix. The chemical composition of the experimental materials were listed in Table 1.

### 2.2. Experimental Procedure

**Figure 1:**
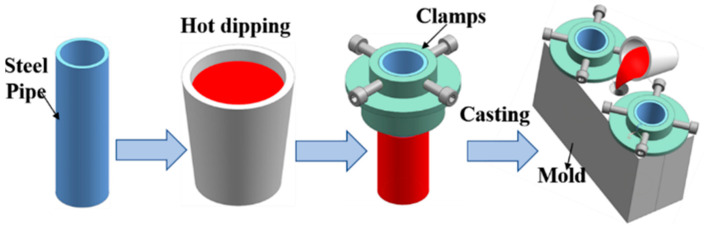
Schematic diagram of bimetallic casting.

The steel substrates had a diameter of 36 mm, a height of 115 mm, and a wall thickness of 3 mm. Before the experiment, the steel substrates were ground by silicon carbide paper and then were immersed in 15 wt.% sodium hydroxide solution at 45 °C for 20 min to remove oil contamination from the steel substrate surface. Next, the steel substrates were immersed in 10 wt.% hydrochloric acid to remove rust from the surface of the steel substrates, and finally were soaked in 5 wt.%K_2_ZrF_6_ solution at 90 °C for 10 min. Hot dipping aluminum was firstly conducted by immersing steel pipe 45 into Al-7Si alloy with different Eu contents melted at 730 °C for 5 min. Subsequently, steel pipe after hot dipped aluminum was quickly put into a metal mold with a preheated temperature of 400 °C, and the Al-7Si alloys melt of the same composition as the hot-dip alloys with a temperature of 730 °C were poured into the metal mold. Al/Steel bimetallic composites were finally obtained after casting solidification. Figure 1 shows a schematic diagram of bimetallic casting.

### 2.3. Microstructural Characterizations

The interface microstructure of Al/Steel bimetallic castings was observed using JSM-IT800 (JEOL, Tokyo, Japan) scanning electron microscope (SEM) with energy-dispersive X-ray spectroscopy (EDS) and an optical microscopy (OM). Samples were ion-polished by an argon ion polishing instrument (JEOL IB-19530CP) (JEOL, Tokyo, Japan) at 6 kV for 30 min and then analyzed using EBSD analysis (Oxford C-nano) (Oxford, London, UK) for the verification of the phases formed at the Al/Steel bimetallic interface. Electron probe microanalysis (EPMA; JXA-8230) (JEOL, Tokyo, Japan) was performed to analyze the elemental distribution at the Al/steel bimetallic interface. The thickness of the intermetallic compounds at the Al/Steel bimetallic interface was calculated using the Image Pro software. To reduce the error, five SEM photographs of each Eu content and 10 places were measured in each photograph.

### 2.4. Mechanical Characterizations

**Figure 2:**
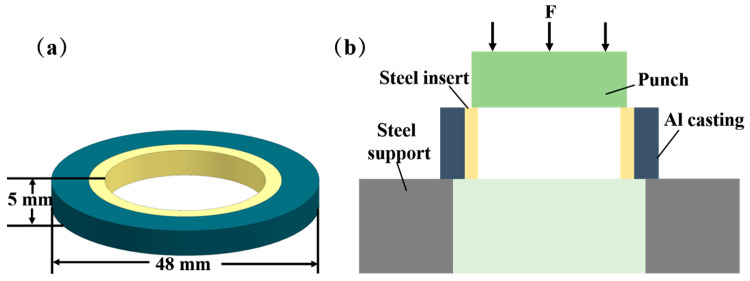
Size and shear diagram of shear specimen: (**a**) Shear specimen size (**b**) Shear diagram.

The shear strength of the specimens was measured using a WDW-300 microcomputer-controlled electronic universal testing machine. As seen in Figure 2, the shear sample was fixed on the testing machine. The indenter was allowed to make contact with the test piece, which was then loaded from top to bottom at a loading speed of 1 mm/min so that the test piece broke along the interface of the Al/Steel composite. To ensure the precision of the test results, five samples were selected for the shear strength test in each group of processes, and the average values were considered as the shear strength of the samples.

## 3. Results

### 3.1. Effect of Eu on Eutectic Si of Al-Si Alloys

**Figure 3:**
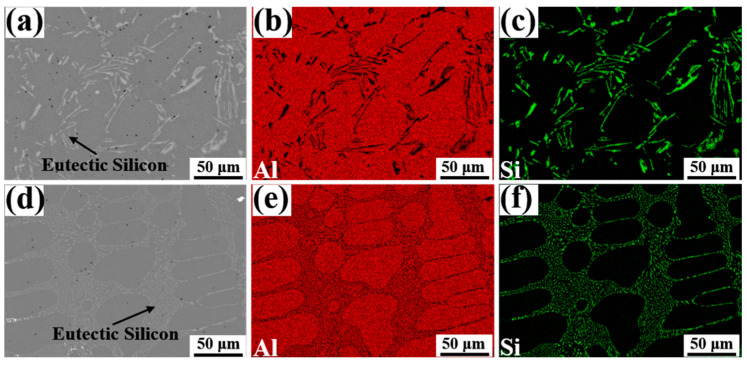
The morphology of eutectic Si and elements distributions of Al and Si elements in Al-7Si alloys with different Eu contents: (**a**–**c**) 0 wt.% Eu; (**d**–**f**) 0.1 wt.% Eu.

Figure 3 reveals the morphology of eutectic Si and elements distributions in Al-7Si alloys with different Eu contents. Due to the small solid solution of Si in Al, the Si element was mainly present in the form of plate-like eutectic silicon in the Al matrix, as indicated in Figure 3a–c, which easily splits the alloy matrix and thus severely weakens its mechanical properties. As seen in Figure 3d–f, after the addition of 0.1 wt.% Eu, the morphology of the eutectic silicon in the alloy changed significantly from plate-like to fine fibers. In addition, the distribution of eutectic silicon became more homogeneous and continuous with each other.

### 3.2. Effect of Eu Contents on Interface Microstructures of Al/Steel Bimetallic Composites

**Figure 4:**
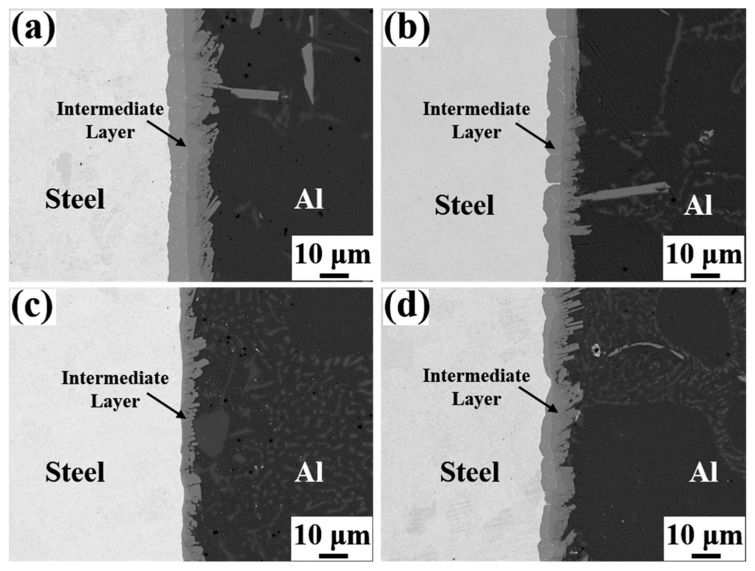
SEM micrographs of Al/Steel bimetallic interfaces with different Eu contents: (**a**) 0 wt.% Eu; (**b**) 0.05 wt.% Eu; (**c**) 0.1 wt.% Eu; (**d**) 0.2 wt.% Eu.

**Figure 5:**
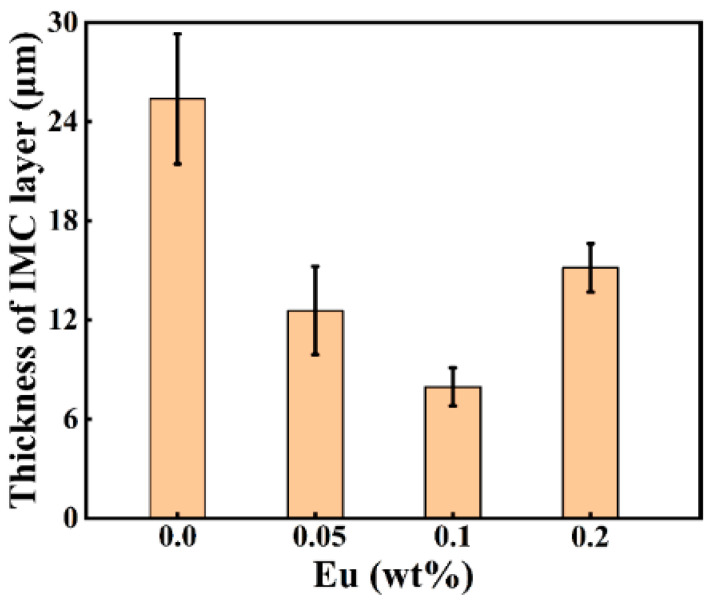
Intermediate layer thickness at the Al/Steel bimetallic interface with different Eu contents.

Figure 4 depicts the SEM micrographs of the Al/Steel interface with different Eu contents, showing a uniform and dense intermediate layer at the interface. As presented in Figure 4a–c, the thickness of the intermetallic compound layer decreased as the Eu content increased from 0 wt.% to 0.1 wt.%, and the intermetallic compound layer near the steel substrate side became gentle. With the Eu content increasing from 0.1 wt.% to 0.2 wt.%, the thickness of the intermetallic compound layer began to increase, and the undulating shape intermetallic compound layer again appeared on the steel substrate side. The measured results of the average thickness of the intermetallic compound layer at different Eu contents are shown in Figure 5. The average thickness of the intermetallic compound layer reached a minimum value of 7.96 μm at 0.1 wt.% Eu.

**Figure 6:**
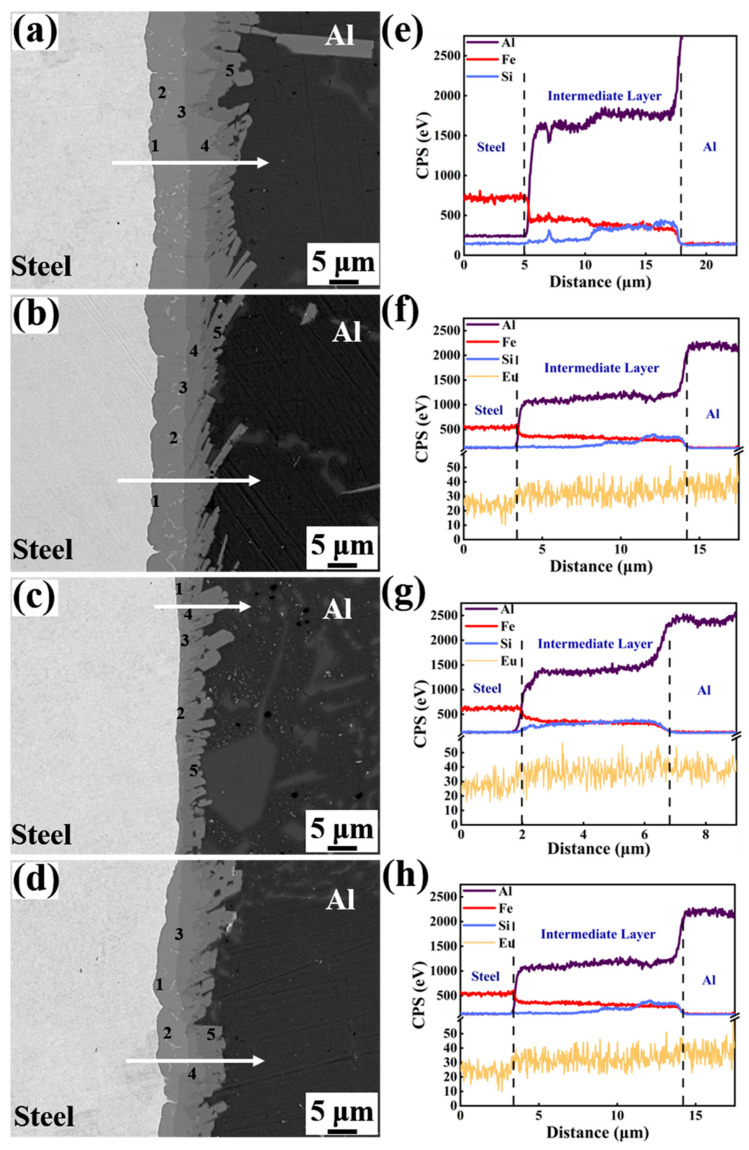
SEM micrographs and EDS line analysis of the Al/Steel bimetallic interface with different Eu contents: (**a**,**e**) 0 wt.% Eu; (**b**,**f**) 0.05 wt.% Eu; (**c**,**g**) 0.1 wt.% Eu; (**d**,**h**) 0.2 wt.% Eu.

Figure 6 displays the magnified SEM micrographs and EDS line analysis of the Al/Steel bimetallic interface with different Eu contents. The obvious delamination was observed in the intermediate layers of bimetallic interfaces with different Eu contents, indicating that there are many kinds of Al-Fe-Si binary or ternary compounds in the interlayer, as displayed in Figure 6a–d. To identify the chemical composition of each point in the interface of Figure 6a–d, Table 2, Table 3, Table 4 and Table 5 show the results of energy dispersive spectrometer (EDS) point analysis in the interface. The Al_5_Fe_2_, τ_1_-(Al, Si)_5_Fe_3_, Al_13_Fe_4_, τ_5_-Al_7_Fe_2_Si, and τ_6_-Al_9_Fe_2_Si_2_ phases were found in the intermetallic layers from the steel matrix to the Al matrix in the Al/steel bimetallic interface. In addition, the thickness of the Al_5_Fe_2_ layer at the interface decreased with the addition of Eu, as seen in Figure 6a–d. The EDS line analysis indicates that Al, Si, Fe, and Eu elements are diffused at the interface and the fluctuation of the element distribution line in the interface becomes gentle after adding Eu, as depicted in Figure 6e–h. At 0.1 wt.% Eu, there was a more sudden drop of Fe in steel side and the Si in Al side was observed to be more than the other conditions, which is because that the Al_5_Fe_2_ and Al_13_Fe_4_ phases are relatively thin, and the intermetallic layer is mainly consisted of τ_5_-Al_7_Fe_2_Si and τ_6_-Al_9_Fe_2_Si_2_ phases.

**Figure 7:**
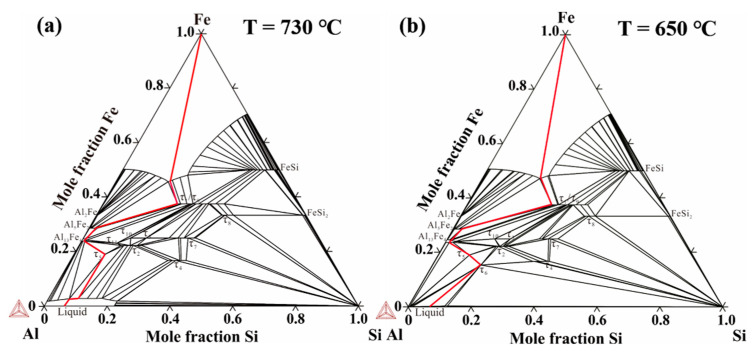
Isothermal sections of Al-Fe-Si system at (**a**) 730 °C and (**b**) 650 °C along with the diffusion path of Al-Si/Fe bimetal.

The thermo-Cal software was used to analyze the phase composition of the Al/Steel bimetallic interface based on the thermodynamic database of the Al-Fe-Si system established by Du et al. [32]. Figure 7 indicates the calculated isothermal sections of the Al-Fe-Si system at 730 °C and 650 °C. It can be seen that there are many kinds of binary and ternary compounds in the Al-Fe-Si system, which the τ_6_-Al_9_Fe_2_Si_2_ phase is formed below 650 °C. According to the experimental results, the diffusion paths of Al-7Si alloy and 45 steel were plotted with red lines in the isothermal sections of the Al-Fe-Si system at 730 °C and 650 °C calculated by Thermo-Calc software, as illustrated in Figure 7. The τ_6_-Al_9_Fe_2_Si_2_, τ_5_-Al_7_Fe_2_Si, Al_13_Fe_4_, Al_5_Fe_2_, and τ_1_-(Al, Si)_5_Fe_3_ phases are formed from Al side to steel side due to the mutual diffusion of elements, which is consistent with the results of SEM and EDS point analysis in Figure 6 and Table 2, Table 3, Table 4 and Table 5.

**Figure 8:**
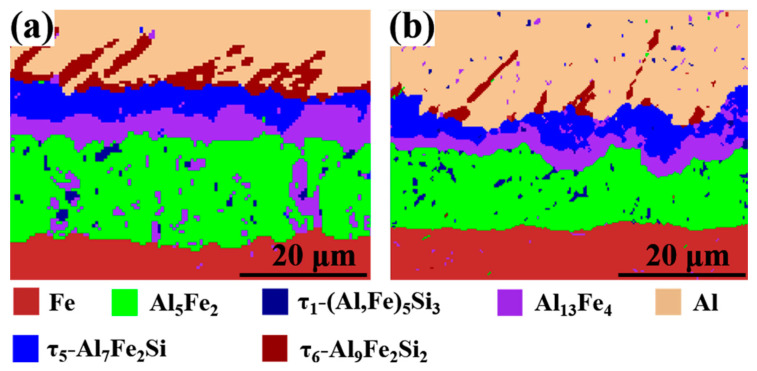
EBSD phase distribution diagram of the intermetallic layer with different Eu contents: (**a**) 0 wt.% Eu; (**b**) 0.2 wt.% Eu.

In order to accurately identify the intermetallic phase [33,34,35,36,37], the EBSD phase distribution diagram of the intermetallic layer with different Eu contents are shown in Figure 8. It can be determined that the phases from the steel side to the Al side were Al_5_Fe_2_ layer, dispersed τ_1_-(Al, Si)_5_Fe_3_ in Al_5_Fe_2_, Al_13_Fe_4_ layer, τ_5_-Al_7_Fe_2_Si layer, and τ_6_-Al_9_Fe_2_Si_2_ layer in the Al/Steel bimetallic interface layer without Eu addition and with 0.2 wt.% Eu content. The phase type of the intermetallic compound layer did not change with the addition of rare earth Eu, which is also consistent with the results of SEM and EDS point analysis in Figure 6 and Table 2, Table 3, Table 4 and Table 5.

### 3.3. Effect Mechanism of Eu on Liquid-Solid Al/Steel Bimetallic Interface

**Figure 9:**
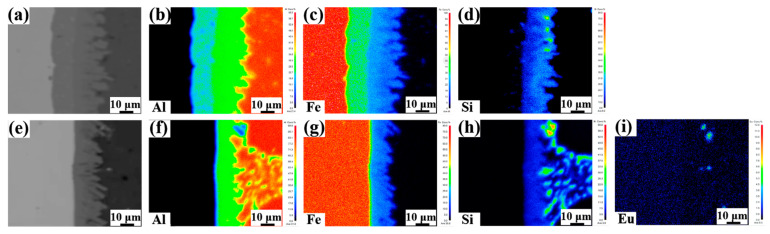
Elements distributions of the Al/Steel interface with the addition of 0 wt.% and 0.1 wt.% Eu contents: (**a**,**e**) The morphology of the interface; (**b**,**f**) Al; (**c**,**g**) Fe; (**d**,**h**) Si; (**i**) Eu.

Figure 9 shows the Al, Fe, Si, and Eu elements distribution of the Al/Steel bimetallic interface with the addition of 0 wt.% and 0.1 wt.% Eu contents. It can be from Figure 9b,c,f,g, that the Fe content decreased from the steel matrix to the aluminum matrix, which was the highest in the Al_5_Fe_2_ phase of the intermetallic compound layer. The Al content decreased gradually from the aluminum matrix to the steel matrix, which was the highest in the ternary phases (τ_6_-Al_9_Fe_2_Si_2_, τ_5_-Al_7_Fe_2_Si,) of the intermetallic compound layer. However, after the addition of 0.1 wt.% Eu, the Fe, and Al element distribution area tended to narrow in the interface. The Si element was mainly distributed in ternary compounds (τ_6_-Al_9_Fe_2_Si_2_, τ_5_-Al_7_Fe_2_Si, τ_1_-(Al, Si)_5_Fe_3_) and the aluminum matrix, as seen in Figure 9d,h. It should be pointed out that the Eu element was found to be mainly distributed at the front of the ternary compound (τ_6_-Al_9_Fe_2_Si_2_) and steel matrix, as displayed in Figure 9i.

**Figure 10:**
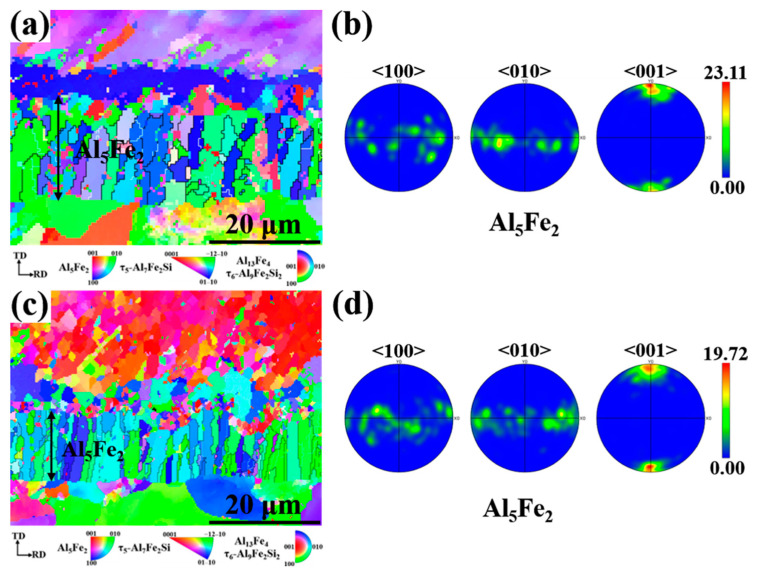
Grain orientation distribution map of bimetallic interface and the grain boundary and polar pole diagram of Al5Fe2 phase: (**a,b**) 0 wt.% Eu; (**c,d**) 0.2 wt.% Eu.

**Figure 11:**
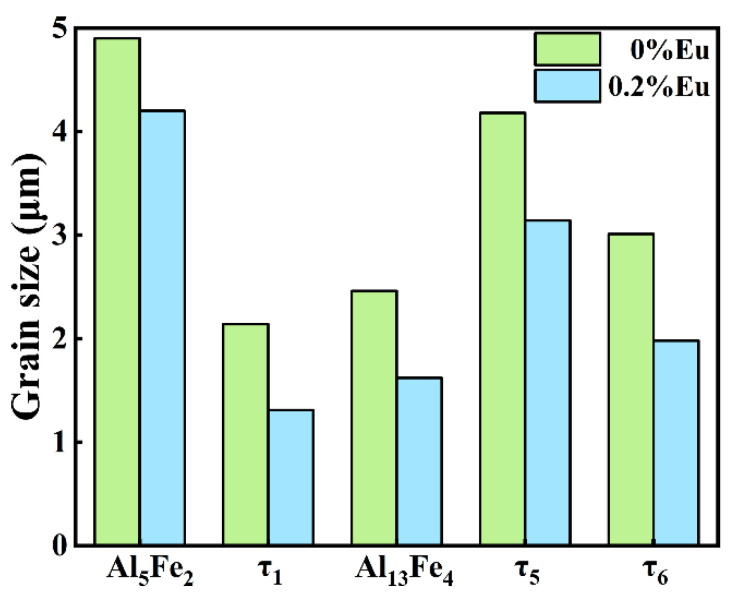
The grain size of each phase in the intermetallic compound layer.

Since the hard and brittle Al_5_Fe_2_ phase has the greatest influence on the interfacial properties, the Al_5_Fe_2_ phase in different Eu contents was analyzed by EBSD. The grain orientation diagrams of the different phases and the polar pole diagram of the Al_5_Fe_2_ phase in the Al/steel bimetallic interfacial layer at 0 wt.% and 0.2 wt.% Eu contents were plotted using Aztec Crystal software, as shown in Figure 10. It can be seen from Figure 10a,c that most of the Al_5_Fe_2_ grains at the interface grew in the form of columnar grains perpendicular to the bimetallic interface. Figure 10b shows that the Al_5_Fe_2_ grains exhibited obvious preferential orientation in the <001> direction, and the polar density was 23.11. With the addition of 0.2 wt.% Eu, the Al_5_Fe_2_ grains also had a preferred orientation in the <001> direction, with a polar density of 19.72, as seen in Figure 10d. The addition of the Eu element does not change the preferential orientation of the Al_5_Fe_2_ phase in the direction but reduces the extreme density value of the preferred orientation. Figure 11 illustrates the grain size of each phase in the intermetallic compound layer at 0 wt.% and 0.2 wt.% Eu contents according to EBSD statistics. The results show that the grain sizes of each phase were refined in the interface reaction layer at 0.2 wt.% Eu.

From the above results, it can be seen that the addition of Eu substantially reduced the thickness of the Al_5_Fe_2_ phase in the Al/steel bimetallic interface reaction layer. The results of existing studies [38,39] show that the growth of the Al_5_Fe_2_ phase in the reaction layer is typically controlled by atomic diffusion from the Al side to the steel side. The atomic stacking model of Al_5_Fe_2_ is a face-centered orthogonal structure, and there are many vacancies and gaps in the c-axis <001> direction of its crystal lattice. Al atoms preferentially diffuse through the c-axis direction of the Al_5_Fe_2_ phase, resulting in the rapid growth of the Al_5_Fe_2_ phase [40,41,42]. The most probable reason for the lower thickness and polar density of the Al_5_Fe_2_ phase is that Eu may inhibit the diffusion of Al atoms along the c-axis direction of the Al_5_Fe_2_ phase.

As the τ_6_-Al_9_Fe_2_Si_2_ phase is formed below 650 °C, the formation of Al_13_Fe_4_, τ_5_-Al_7_Fe_2_Si, and τ_6_-Al_9_Fe_2_Si_2_ layers is affected by the Fe diffusion from the steel side to the Al side and the solidification of τ_6_-Al_9_Fe_2_Si_2_ phase. Due to the small solid solution of element Eu in the intermetallic compound during solidification, Eu will be pushed out at the front of the binary or ternary compound layers (Al_13_Fe_4_, τ_5_-Al_7_Fe_2_Si, and τ_6_-Al_9_Fe_2_Si_2_), which can be proved by the enrichment of Eu element at the front of the ternary compound layer (τ_6_-Al_9_Fe_2_Si_2_) near the aluminum side in Figure 9i. The enrichment of the Eu element at the front of the binary or ternary compound layers may hinder the growth of Al_13_Fe_4_, τ_5_-Al_7_Fe_2_Si, and τ_6_-Al_9_Fe_2_Si_2_ phases, resulting in the thinner Fe element distribution area on the Al side. The thickness of the intermetallic compound layer began to increase at 0.2 wt.% Eu. Eu element may have an larger diffusion rate from Al side to steel side in this condition, leading to the lower Eu content in Al side, which may weaken the ability to restrict the diffusion of Al in C-axis of Al_5_Fe_2_ phase and the growth of Al_13_Fe_4_, τ_5_-Al_7_Fe_2_Si, and τ_6_-Al_9_Fe_2_Si_2_ phases.

### 3.4. Mechanical Properties of the Al/Steel Bimetal

**Figure 12:**
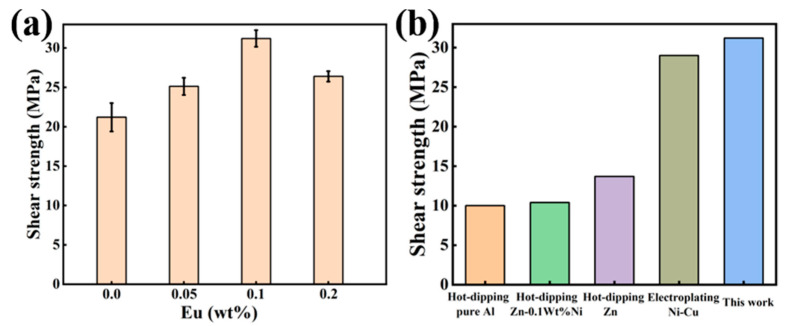
(**a**) Shear strength of Al/Steel bimetal with different Eu contents; (**b**) The comparison of shear strength results at the Al/Steel bimetal interface for different sur-face treatment.

Figure 12a illustrates the shear strength of Al/Steel bimetal specimens added with different Eu contents. The results indicate that when the Eu content is 0.1 wt.%, the shear strength of the bimetallic specimen is the highest, which is 31.21MPa and 47% higher than that of the bimetallic specimen without Eu addition. However, when the Eu content was increased to 0.2 wt.%, the shear strength of the samples decreased to 26.39MPa. Figure 12b shows the comparison of shear strength results of the Al/Steel bimetal interface under different surface treatments [14,43,44], and the newly developed Al/Steel bimetal of this study has the highest shear strength values.

**Figure 13:**
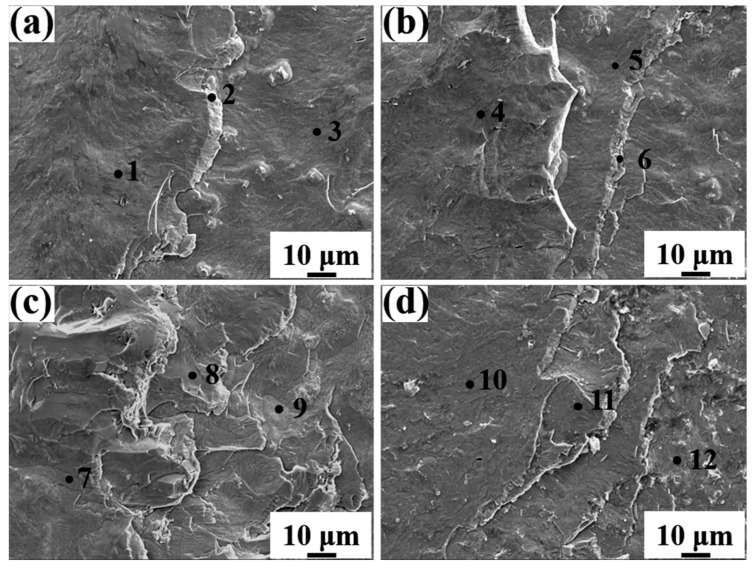
Shear fracture morphology at steel matrix side with different Eu contents: (**a**) 0 wt.% Eu; (**b**) 0.05 wt.% Eu; (**c**) 0.1 wt.% Eu; (**d**) 0.2 wt.% Eu.

Figure 13 is the SEM images of fracture morphologies of Al/steel bimetal samples on the steel substrate side with different Eu contents. It can be seen from the figure that there are cleavage steps and tear edges in the fracture morphology of the samples, and no obvious plastic deformation occurs, indicating that the samples belong to brittle fractures. As illustrated in Table 6, EDS results show that the fracture structure is mainly the Al_5_Fe_2_ phase. As the Eu content increased from 0 wt.% to 0.1 wt.%, the number of tear edges at the fracture increased, as seen in Figure 13a–c. However, as seen in Figure 13d, when the Eu content increased to 0.2 wt.%, the number of tear edges on the fracture surface begins to decrease. The possible reasons for improving the mechanical properties are as follows: (1) The Al_5_Fe_2_ phase layer near the steel side is gentle and has a refiner grain with the addition of 0.1 wt.% Eu. (2) The reaction layer’s thickness is thinner with the addition of 0.1 wt.% Eu. (3) The modification of eutectic Si in the Al-Si alloy matrix after Eu addition. The above reasons can decrease the stress concentration in the reaction layer and improve the shear strength of the Al/steel bimetal.

## 4. Conclusions

(1) With the addition of 0.1 wt.% Eu, the morphology of eutectic silicon changed from plate-like to fine fibers with a more uniform distribution in the Al-Si alloy.
(2) The Al/steel bimetallic interfacial reaction layer was composed of Al_5_Fe_2_, τ_1_-(Al, Si)_5_Fe_3_, Al_13_Fe_4_, τ_5_-Al_7_Fe_2_Si, and τ_6_-Al_9_Fe_2_Si_2_ phases. The intermetallic compound species in the reaction layer were unaffected by the addition of Eu.
(3) When the Eu content is 0.1 wt.%, the average thickness of the reaction layer and the Al_5_Fe_2_ layer decreased to the minimum value. In addition, there was a more sudden drop of Fe in steel side and the Si in Al side was observed to be more than the other conditions.
(4) The thickness of Al and Fe elements distribution in the reaction layer decreased as the content of Eu reached 0.1 wt.%. Eu was mainly enriched in the front of the ternary compound layer (τ_6_-Al_9_Fe_2_Si_2_) near the Al side and steel matrix.
(5) The addition of the element Eu did not change the preferred orientation of the Al_5_Fe_2_, τ_1_-(Al, Si)_5_Fe_3_, Al_13_Fe_4_, τ_5_-Al_7_Fe_2_Si, and τ_6_-Al_9_Fe_2_Si_2_ phases, but refined the grain size of each phase in the interfacial reaction layer.
(6) The highest shear strength of bimetallic specimens was obtained when the Eu content was 0.1 wt.%, which was 47% higher than that of bimetallic specimens without Eu addition.

## Figures and Tables

**Table 1:** Chemical compositions of the experimental materials (wt.%).

Alloys	Compositions							
	Al	Si	Eu	Fe	Cu	C	Mn	Cr
45 steel	-	0.24	-	Bal.	0.21	0.45	0.57	0.17
Al-7Si	Bal.	6.97	0	0.07	<0.01	-	<0.01	-
Al-7Si-0.05Eu	Bal.	6.98	0.047	0.06	<0.01	-	<0.01	-
Al-7Si-0.1Eu	Bal.	7.03	0.09	0.06	<0.01	-	<0.01	-
Al-7Si-0.2Eu	Bal.	6.95	0.198	0.08	<0.01	-	<0.01	-

**Table 2:** Energy spectrum analysis results of each point in Figure 6a.

Points	Compositions (at%)			Possible Phase
	Al	Fe	Si	
1	68.62	28.74	2.64	Al_5_Fe_2_
2	48.18	37.71	14.11	τ_1_-(Al, Fe)_5_Si_3_
3	73.93	23.34	2.73	Al_13_Fe_4_
4	68.95	20.86	10.19	τ_5_-Al_7_Fe_2_Si
5	70.69	16.03	13.28	τ_6_-Al_9_Fe_2_Si_2_

**Table 3:** Energy spectrum analysis results of each point in Figure 6b.

Points	Compositions (at%)				Possible Phase
	Al	Fe	Si	Eu	
1	69.16	29.44	1.39	0.01	Al_5_Fe_2_
2	46.37	39.03	14.58	0.02	τ_1_-(Al, Fe)_5_Si_3_
3	71.01	24.34	3.99	0.06	Al_13_Fe_4_
4	67.09	21.71	11.12	0.08	τ_5_-Al_7_Fe_2_Si
5	69.49	15.24	15.08	0.19	τ_6_-Al_9_Fe_2_Si_2_

**Table 4:** Energy spectrum analysis results of each point in Figure 6c.

Points	Compositions (at%)				Possible Phase
	Al	Fe	Si	Eu	
1	67.11	29.75	3.14	0.00	Al_5_Fe_2_
2	53.89	34.95	11.15	0.01	τ_1_-(Al, Fe)_5_Si_3_
3	70.74	25.67	3.54	0.05	Al_13_Fe_4_
4	68.37	20.64	10.89	0.1	τ_5_-Al_7_Fe_2_Si
5	67.48	15.56	16.32	0.64	τ_6_-Al_9_Fe_2_Si_2_

**Table 5:** Energy spectrum analysis results of each point in Figure 6d.

Points	Compositions (at%)				Possible Phase
	Al	Fe	Si	Eu	
1	68.18	29.02	2.79	0.01	Al_5_Fe_2_
2	45.51	15.25	39.18	0.06	τ_1_-(Al, Fe)_5_Si_3_
3	73.95	23.57	2.41	0.07	Al_13_Fe_4_
4	67.69	20.94	11.19	0.18	τ_5_-Al_7_Fe_2_Si
5	65.88	16.91	16.78	0.43	τ_6_-Al_9_Fe_2_Si_2_

**Table 6:** Energy spectrum analysis results of each point in Figure 13.

Points	Compositions (at%)				Phase
	Al	Fe	Si	Eu	
1	70.59	27.51	1.90	0.00	Al_5_Fe_2_
2	69.09	28.29	2.62	0.00	Al_5_Fe_2_
3	69.81	27.85	2.34	0.00	Al_5_Fe_2_
4	69.58	28.07	2.18	0.17	Al_5_Fe_2_
5	69.20	28.10	2.63	0.07	Al_5_Fe_2_
6	69.64	28.90	1.31	0.15	Al_5_Fe_2_
7	67.36	29.93	2.50	0.21	Al_5_Fe_2_
8	68.34	27.34	3.86	0.46	Al_5_Fe_2_
9	70.61	27.87	1.39	0.13	Al_5_Fe_2_
10	69.76	28.44	1.75	0.06	Al_5_Fe_2_
11	70.51	28.09	1.29	0.11	Al_5_Fe_2_
12	67.92	29.14	2.86	0.08	Al_5_Fe_2_

## Data Availability

The data presented in this study are available on request from the corresponding author.
